# Monocular Presentation Attenuates Change Blindness During the Use of Augmented Reality

**DOI:** 10.3389/fpsyg.2019.01688

**Published:** 2019-07-31

**Authors:** Akihiko Kitamura, Yasunori Kinosada, Kazumitsu Shinohara

**Affiliations:** ^1^Applied Cognitive Psychology Lab, Graduate School of Human Sciences, Osaka University, Suita, Japan; ^2^Faculty of Informatics, Shizuoka Institute of Science and Technology, Fukuroi, Japan

**Keywords:** change blindness, augmented reality, binocular rivalry, monocular presentation, visual attention, human interface

## Abstract

Augmented reality (AR) is an emerging technology in which information is superimposed onto the real world directly in front of observers. AR images may behave as distractors because they are inside the observer’s field of view and may cause observers to overlook important information in the real world. This kind of overlooking of events or objects is known as “change blindness.” In change blindness, a distractor may cause someone to overlook a change between an original image and a modified image. In the present study, we investigated whether change blindness occurs when AR is used and whether the AR presentation method influences change blindness. An AR image was presented binocularly or monocularly as a distractor in a typical flicker paradigm. In the binocular presentation, the AR image was presented to the both of the participants’ eyes, so, it was not different from the typical flicker paradigm. By contrast, in the monocular presentation, the AR image was presented to only one eye. Therefore, it was hypothesized that if participants could observe the real-world image through the eye to which the AR image was not presented, change blindness would be avoided because the moment of change itself could be observed. In addition, the luminance of the AR image was expected to influence the ease to observe the real world because the AR image is somewhat translucent. Hence, the AR distractor had three luminance conditions (high, medium, and low), and we compared how many alternations were needed to detect changes among the conditions. Result revealed that more alternations were needed in the binocular presentation and in the high luminance condition. However, in all luminance conditions in the monocular presentation, the number of alternations needed to detect the change was not significantly different from that when the AR distractor was not presented. This result indicates that the monocular presentation could attenuate change blindness, and this might be because the observers’ visual attention is attracted to the location where the change has occurred automatically.

## Introduction

### Spread of Augmented Reality

Augmented reality (AR), in which the information is superimposed onto the real world directly in front of an observer, is one of the most promising information presentation technologies to emerge in recent decades (Azuma, 1997; Azuma et al., 2001; Chatzopoulos et al., 2017). AR is very intuitive, and users do not have to shift their gaze from in front of them, so AR is expected to enhance usability and safety. For example, in a driving scenario, virtual arrows and warnings can be presented onto a road in the real world to navigate users and to avoid traffic accidents (Rusch et al., 2013; Schwarz and Fastenmeier, 2017; Uchida et al., 2017; Schömig et al., 2018). Moreover, AR can be used for supporting medical surgery (Shuhaiber, 2004; Dixon et al., 2013; Castillo and Olga, 2016), presenting historical sites (Ikeuchi, 2013), and enhancing entertainment-based applications (Joseph and Armstrong, 2016).

However, AR has not only tremendous merits but also some problems. AR superimposes information onto the real world directly, which means that there is a risk that AR information presented over real-world objects will prevent users from observing those objects (Kitamura et al., 2014, 2015). This characteristic of AR causes poor visibility of objects and decreases the performance of tasks conducted in the real world.

To address this problem, monocular AR presentation has been proposed (Sasaki et al., 2010; Kitamura et al., 2014, 2015). AR images can be presented binocularly (i.e., to both of the observer’s eyes) or monocularly (i.e., to one of the observer’s eyes). The binocular AR presentation prevents the user from unobstructedly observing the real world in either eye. On the other hand, the monocular AR presentation allows the user to observe the real world through the eye to which the AR image is not presented. As a result, users can observe the real world more easily and can perform real-world tasks better than in the binocular AR presentation (Kitamura et al., 2015).

### Relationship Between AR and Change Blindness

Nevertheless, although the advantage of the monocular AR presentation was revealed in previous studies, AR has been barely researched in psychology because it is just emerging and has been studied mainly in computer science and engineering. One problematic psychological phenomenon that may occur in actual AR use is “change blindness” (Levin and Simons, 1997; Rensink et al., 1997; Simons and Levin, 1998; O’Regan et al., 1999; Galpin et al., 2009; Jensen et al., 2011; Paffen et al., 2012; Smith et al., 2013; Graham et al., 2018; Murphy and Murphy, 2018). A change between an original image and a modified image is easy to find when they are presented sequentially. However, the change becomes extremely difficult to detect once a blank stimulus is displayed between the original and modified images. Change blindness is a very robust phenomenon that occurs in not only laboratories but also more real-world scenarios (Levin and Simons, 1997; Simons and Levin, 1998; Smith et al., 2013; Graham et al., 2018; Murphy and Murphy, 2018). The phenomenon is so counterintuitive (Levin et al., 2000) that subjectively the observer often feels surprised at missing such an obvious change (Jensen et al., 2011).

One of the most typical experimental procedures for change blindness is the “flicker paradigm” (Rensink et al., 1997; Noë et al., 2000; Jensen et al., 2011). In the flicker paradigm, an original image (A), a modified image (A′), and a blank are used as experimental stimuli and presented as follow: A → blank → A′ → blank → A……. In this procedure, participants can detect the change between two images at the end in most trials but need some alternations to do so. Furthermore, they sometimes cannot find the change even if 1 min elapses after the trial has started.

There are two major explanations of why change blindness occurs. One claims that attention must be directed to the place the change occurs at the moment the change occurs to form robust visual representation (Rensink, 2002). The other emphasizes that visual representation is stored in visual memory even after attention is diverted to another place and can be used to detect the change (Hollingworth and Henderson, 2002; Hollingworth, 2004). According to Nakashima and Yokosawa (2012, 2018), this apparent difference between the two theories depends on the experimental procedure. When stimulus displays are alternated mutually many times, sequential visual information processing is needed like the flicker paradigm, so the former theory explains the change blindness well. Therefore, the former one suits the experimental procedure and objectives in the present study; hence, it is introduced in detail below.

This theory is called “coherence theory” (Rensink, 2002) and proposes three stages of the relationship between the visual attention and representation that is required to detect the change. In the first stage, focused attention is not directed to the place. In this stage, the low-level visual information is processed rapidly and in parallel. The visual information structure is treated as a “proto-object” and is sophisticated enough to provide observes some information from the visual field. However, the visual information structure has only limited spatial and temporal coherence, which is to say, it is very volatile and easily replaced by new stimuli at the same location. In the second stage, focused attention is directed to a particular area or particular objects. Visual attention has limited capacity, hence, only several objects can be chosen. However, the objects can gain robust visual representation. In this stage, objects’ properties are pooled into a single collection point from the proto-objects, and this pooling state is called the “nexus.” Attention engages maintaining feedback and feedforward links between the proto-objects and nexus. The feedback links are involved in collecting properties of objects, and the feedforward links are involved in stabilizing the proto-objects. Coherent forms of the proto-object are held in this stage. In the third stage, attention is removed from the objects. In this stage, the links maintained by attention are broken, and coherent forms of properties cannot be held. This means the visual representation of the object becomes volatile and easily replaced again. According to coherence theory, to detect the change, attention must be directed to the place the change occurs at the moment the change occurs, to compare the visual representation created by the input from before the change with the input from after the change. Otherwise, the change will be overlooked even if attention is directed there, because the coherence collapses again without attention (Rensink, 2002; Nakashima and Yokosawa, 2012, 2018).

The flicker paradigm and coherence theory imply that AR presentation may lead to change blindness. For example, in a driving scenario, an AR image may be presented in front of a driver. In this situation, a child may jump into the road simultaneously as the AR image is presented. This situation is very similar to the flicker paradigm procedure: the situation before the AR image is presented is an original image, the AR image itself is a distractor, and the situation after the emergence of the child is a modified image. Therefore, the driver may overlook the child because the AR image behaves as a distractor that prevents the driver from directing his/her attention to where the child is, and the result may be a fatal accident. Because of the concept of AR, the AR image is always inside the field of view of a user (Azuma, 1997; Azuma et al., 2001; Chatzopoulos et al., 2017). Therefore, it is difficult to prevent the AR image from behaving as a distractor.

### Related Work

As mentioned above, the flicker paradigm and the situations in which AR is used share some similarities. Hence, change blindness when the AR is used should be investigated. In related work, Dixon et al. (2013) investigated how AR presentation influences “inattentional blindness.” Inattentional blindness (Mack and Rock, 2000; Jensen, et al., 2011) is the phenomenon in which observers miss some distinct stimulus when they concentrate on another task, especially a visual task (Simons and Chabris, 1999). This overlooking occurs because of lack of attention to the object or place, so there are some similarities to change blindness. Dixon et al. (2013) presented AR images to support medical surgery training, and during the training, some critical events occurred. They revealed that participants missed the critical events more often in the condition in which the AR information was superimposed onto the body image than in the control (no AR information) condition. This result indicates that AR information attracts attention and causes practical problems.

However, in their study, Dixon et al. (2013) investigated only inattentional blindness not change blindness. Even though participants miss critical events because their attention is distributed elsewhere in both inattentional blindness and change blindness, their experimental procedures have some differences. In a typical inattentional blindness task, participants concentrate on the other main task and do not expect something unusual to occur; hence, they do not distribute their attention to seeking the event actively. In addition, no blank or distractor is presented during the main task, so participants can observe just the presentation of the event. On the other hand, in the typical flicker change blindness task, participants are fully aware that a change will occur, so they actively look out for something unusual. In addition, distractors or blanks are presented with the change, unlike in an inattentional blindness task. Therefore, the attention distribution strategies and presentation method of event and distractors are vastly different between inattentional blindness and change blindness. These differences mean that inattentional blindness and change blindness occur in different situations and for different reasons in actual AR use. For example, if a driver concentrates on reading information in AR, the driver may overlook a pedestrian because of not paying enough attention to the road. This is related to inattentional blindness. On the other hand, even if a driver concentrates on the driving task, the driver may still overlook the pedestrian if AR images pop up for notification. This situation is related to change blindness. Therefore, change blindness, not inattentional blindness, in AR use should be investigated. In addition, the difference between the binocular and monocular presentations was not addressed by Dixon et al. (2013), whereas the comparison between the observation conditions is one of the main topics in the present study.

Steinicke and Hinrichs (2011) researched change blindness in virtual reality (VR) environments. They used a head-mounted display (HMD) to present stimuli and distractors for change blindness. They compared a monoscopic flicker condition, which is almost the same as the typical flicker paradigm, with a stereoscopic flicker condition, in which the stimuli and distractor were presented to the right eye and left eye in turn. However, the time course was the same as in the monoscopic flicker condition, so participants could not observe the moment of the change. In addition, there was a phase-shifted flicker condition, in which the stimuli were presented to the right eye and left eye in turn. As a result, stimuli were always presented to one of their eyes, so participants could observe the change itself. The result revealed that the alternation to detect the change was less in the phase-shifted flicker condition because participants could observe stimuli when the change occurred. This indicates that the observation of the period of change is very important to detect the change, and the binocular observation of the stimuli is not needed to detect it. Nevertheless, although Steinicke and Hinrichs (2011) investigated the difference between the binocular presentation (stereoscopic condition) and monocular presentation (phase-shifted condition) in change blindness, the situation is somewhat far from that in the AR presentation, because in optical see-through AR, AR images are somewhat translucent. Therefore, users can see the real world through the AR images even in the binocular condition. In addition, in actual AR use, the eye to which the AR image is presented hardly ever switches, like it does in a phase shifted condition.

Therefore, comparison between the binocular and monocular AR presentations in change blindness has still not been investigated enough.

### Objectives of the Present Study

To tackle this problem, the present study has two objectives: (1) to investigate whether change blindness occurs during AR use and (2) to investigate whether the AR presentation method influences change blindness. For the second objective, we focused on the observation condition (binocular and monocular) and luminance (high, medium, and low) of the AR image as the factors that influence change blindness in AR use. The reasons we chose these as important factors are explained as below.

The difference between the binocular and monocular presentations might lead to different result in terms of change blindness. In the binocular presentation, the AR image is presented to both of the observer’s eyes, so it is a very similar situation to the typical flicker paradigm. This means that change blindness cannot be avoided in the binocular presentation. On the other hand, in the monocular presentation, the AR image is presented to only one of the observer’s eyes, so the observer can see the real world through the eye to which the AR image is not presented. In the flicker paradigm, if there is no distractor between the original and modified images, an observer hardly ever misses the change. This is because an abrupt onset of a stimulus, like the change, attracts attention automatically and forcibly (Posner, 1980; Yantis and Jonides, 1984; Wright and Ward, 2008), hence, the image before the change is easy to compare with that after the change even in the flicker paradigm. Therefore, in the monocular AR presentation, in the one eye, the change is very easy to detect, and in the other eye, the situation is similar to the typical flicker paradigm. Therefore, if the observer can choose the information from the eye to which the AR image is not presented, change blindness might be avoided in the monocular presentation.

When extremely different images are presented to each eye, like in the monocular AR presentation, the images compete for dominance, and when one image is perceived, the other image is suppressed. This ongoing perceptual alternation is known as binocular rivalry (Levelt, 1966; Kovács et al., 1996; Chong and Blake, 2006; Hancock and Andrews, 2007; Paffen and Alais, 2011; Zhang et al., 2011). In binocular rivalry, the characteristics of the stimuli help determine which stimulus tends to be dominant (Levelt, 1966; Paffen and Alais, 2011). For example, high contrast, high luminance, or dynamic stimuli tend to be more dominant than low contrast, low luminance, or static stimuli. In summary, stimuli having high intensity tend to be dominant.

In the typical change blindness flicker paradigm, the original and modified images are real-world pictures, whereas a distractor or blank is static. In the present study, we replicated this setting; hence, the AR image as a distractor was a static gray rectangle. Given the characteristics of binocular rivalry (Levelt, 1966), the AR distractor in the monocular presentation would be suppressed because the counterpart is a real-world picture, which is meaningful and has tremendous edges and colors. Therefore, it could be thought that the participant would be able to observe the information from the eye to which the AR image is not presented in the monocular presentation. Thus, it is supposed that the AR image as a distractor may behave differently in the binocular and monocular presentation, resulting in change blindness hardly occurring in the monocular presentation due to the characteristics of binocular rivalry while occurring in the binocular presentation.

In addition, given actual AR use scenarios, the luminance of AR images might influence change blindness. As mentioned above, in optical see-through AR, AR images are superimposed onto the real world directly but are somewhat translucent. Therefore, users can see the real world through the AR images. The higher the luminance of the AR, the higher the visibility of the AR image itself. Of course, higher visibility is desirable for actual use. However, higher visibility of the AR images means that the images cover the real world more strongly and that the real world becomes less visible (Kitamura et al., 2015). Therefore, a high luminance AR image might behave as a stronger distractor than a low luminance AR image, and as a result, change blindness might occur more frequently.

Moreover, as mentioned above, the AR distractor would not evoke change blindness in the monocular presentation because the AR perception would be suppressed in the flicker paradigm due to binocular rivalry. Therefore, the luminance might not influence how often change blindness occurs in the monocular presentation. In other words, in the monocular presentation, change blindness would not occur in any luminance conditions.

In summary, we investigated two hypotheses. The first hypothesis is that change blindness would occur less in the monocular presentation than in the binocular presentation, because in the monocular presentation, participants seem to be able to see the real world through the eye to which the AR image is not presented. The second hypothesis is that, in the binocular presentation, change blindness would occur less frequently in the low luminance condition than in the high condition, because the visibility of the real world would be higher in the low luminance condition.

For both hypotheses, to investigate whether change blindness occurs or not, monocular and binocular presentations must be compared with a situation in which change blindness does not occur. Hence, participants were assigned one of three observation conditions: binocular, monocular, or none. In the none condition, no distractor appeared between the original and modified images. In this condition, participants detect the change easily, and change blindness hardly ever occurs. We compared the binocular and monocular conditions with the none condition.

## Experiments

All these experiments were approved by the Behavioral Research Ethics Committee of the Osaka University School of Human Sciences. Written informed consent was obtained from all participants. No participants were under the age of 16. After the experiment was finished, they were paid money for their participation.

### Preliminary Experiment

In the present study, the observation condition (binocular, monocular, and none) was a between design, and the luminance condition (high, medium, and low) was a within design. In change blindness, the same stimuli image pairs cannot be used twice for a particular participant, because once participants have seen a pair of images, they learn where the change occurs (Takahashi and Watanabe, 2008). Therefore, all stimuli pairs have to be different for the luminance condition because it is a within design. However, it is unfair if the stimulus set in one condition consists of trials in which the change is easier to detect than in trials in the other conditions (Rensink et al., 1997; Murphy and Murphy, 2018). Hence, we conducted a preliminary experiment to measure how long it takes to detect the change for 108 pairs of stimuli by using a typical flicker paradigm.

#### Participants

Six students (Male = 3, Female = 3) at Osaka University participated in the preliminary experiment. Their mean age was 21.67 [standard deviation (SD) = 0.75] years old. All participants had normal or corrected-to-normal vision (at least 0.5 as binocular decimal visual acuity). By using Ishihara color test II (24 plates), all participants were certified to have normal color vision.

#### Apparatus

**Figure 1:**
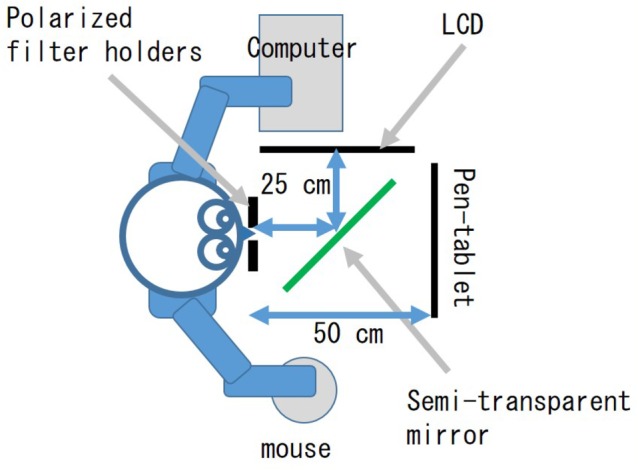
Arrangement of apparatus.

**Figure 2:**
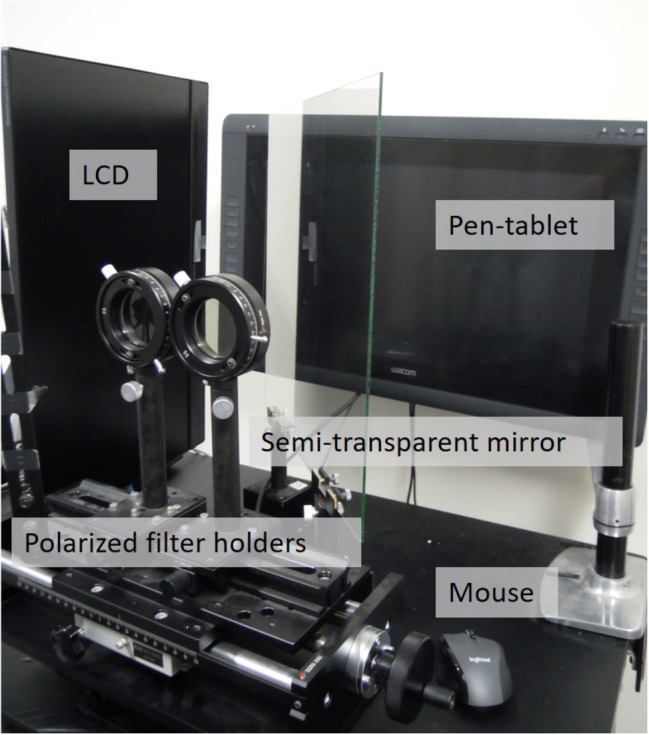
Picture of apparatus.

Polarized filter holders (Sigma koki, PH-50), a semi-transparent mirror, a pen-tablet monitor (WACOM, cintiq 22HD, the resolution was set to 1,680 × 1,050), a liquid crystal display [LCD: Mitsubishi, RDT235WX(BK), AX220 model, the resolution was set to 1,920 × 1,080], a computer (Dell, Inspiron 15 3000 series, OS was Windows 10), and a mouse (Elecom, M-BL09DB) were set as shown in Figures 1, 2. Programs for displaying stimuli and measuring response were created by using Microsoft Visual Studio Community 2015.

Although polarized filter holders and the semi-transparent mirror were not needed to control the stimuli in the preliminary experiment, they were located in front of the participants to make the apparatus arrangement the same as in the main experiment.

The pen-tablet monitor was located at 50 cm from the participants, and all stimuli, both image pairs for the flicker task and a blank, were presented on the monitor.

#### Stimuli

In total, 108 pairs of original and modified images were used. The images were 9.2 cm high and 13.6 cm wide (10.5^°^ × 15.5^°^ as visual angle) real-world pictures without living things. The modified images each contained one of three types of modifications: a change in color, a change in the location of an object, and a disappearing object. During the blank, no stimulus was presented, and the luminance of the display was 0.0 cd/m^2^.

#### Procedure

**Figure 3:**
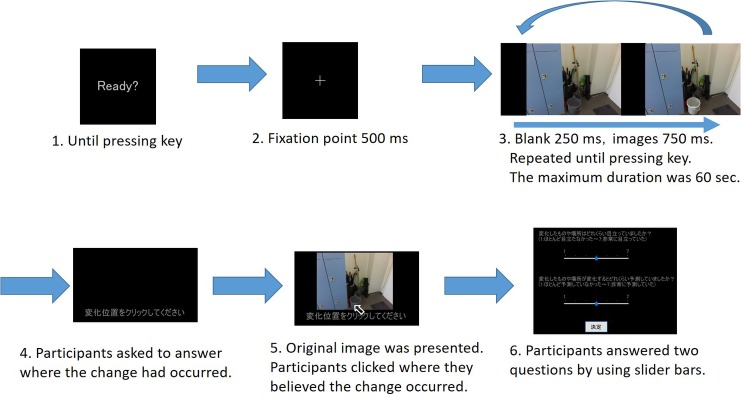
Experimental procedure in preliminary experiment.

In the preliminary experiment, all procedures followed the typical flicker paradigm (see Figure 3). First, “Ready?” appeared on the monitor. When participants pressed the 5 key on the numeric keypad, a cross was presented for 500 ms as a fixation point at the center of the pen-tablet monitor, followed by the blank for 250 ms and then the original image for 750 ms. Next, the blank was presented again for 250 ms, followed by the modified image for 750 ms, the same as the original image. After these presentations, all stimuli were presented repeatedly until participants detected the change and pressed the 5 key to finish the presentation of the stimuli. If participants could not detect the change even after 60 s had elapsed, the presentation of the stimuli finished automatically.

Then, a sentence was presented instructing participants to click the location of the change. Participants pressed the 5 key again, and the original image was presented. Participants clicked where they believed the change had occurred to determine whether they could detect the change correctly. After participants clicked, the original image disappeared, and two questions in Japanese appeared. (1) “How conspicuous was the location or object that changed?” and (2) “How predictable was the location or object that changed?” Participants answered these questions by using sliders below the questions, ranging from one [not at all conspicuous (predictable)] to seven [very conspicuous (predictable)]. After answering, participants clicked the button below the sliders, then the trial finished, and the next trial started.

The 108 pairs of images were separated into three blocks, each consisting of 36 trials. For all participants, the same 36 pairs of images were included in a certain block. However, the order of the trial was random, and the order of blocks was counterbalanced between participants.

#### Results

Thirty-six pairs of images had to be selected for the main experiment, including 12 trials in each luminance condition. Each change condition (color, location, and disappearing) had 12 trials. The criteria for exclusion were as follows. First, if the participants failed to press the button, the trials were excluded (13 trials). Next, if any participant could not detect the change within 60 s or click the location of the change correctly, the pair was regarded as too difficult and excluded (7 and 13 pairs, respectively). The clicked location was classified as an error if participants could not click within 10 pixels from the location or object that changed. Furthermore, if more than two participants responded earlier than 2,250 ms, the pair was regarded as too easy and excluded (24 pairs).

Finally, 12 image pairs for which the average response times of six participants were from 3,250 to 10,250 ms were found in each change condition. We separated these 36 pairs of images into three stimulus sets. All three stimulus sets had similar average response times [5,800 ms (SD = 2,049), 5,802 ms (SD = 1,947), and 5,816 ms (SD = 1,646)]. Therefore, each set had similar difficulty even though the stimulus sets had different contents.

### Main Experiment

In the main experiment, we conducted a flicker paradigm task almost the same as in the preliminary experiment. The difference between the preliminary experiment and the main experiment was that the distractor was presented as an AR gray rectangle to investigate change blindness when AR is used. The AR image was presented binocularly or monocularly and not presented in the none condition. Moreover, we controlled the luminance of the AR image to investigate its influence on change blindness.

#### Participants

Thirty-six students at Osaka University participated in the main experiment. Twelve participants were assigned to each observation condition. In the binocular condition, participants were seven females and five males with a mean age of 23.17 (SD = 5.32). In the monocular condition, participants were six males and six females with a mean age of 21.42 (SD = 1.55). In the none condition, participants were six females and six males with a mean age of 20.75 (SD = 1.09). All participants had normal or corrected-to-normal vision (at least 0.6 as binocular decimal visual acuity). By using Ishihara color test II (24 plates), all participants were certified to have normal color vision.

#### Apparatus

The apparatus and arrangement were the same as in the preliminary experiment.

#### Stimulus

The 36 pairs of the images selected in the preliminary experiment were used. AR images as distractors were gray rectangles. The three luminance conditions of the AR image were high (10.8 cd/m^2^), medium (5.4 cd/m^2^), and low (2.7 cd/m^2^). The size of the AR rectangle was the same in both the original and modified images (10.5° × 15.5° as visual angle). The AR image covered the pairs of the images entirely although participants could still observe the stimuli through the AR image.

#### Procedure

**Figure 4:**
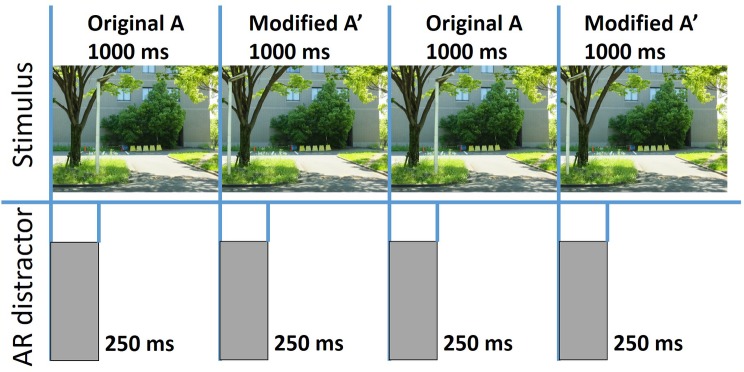
Experimental procedure in main experiment.

The procedure in the main experiment was almost the same as in the preliminary experiment, but the presentation method of the image pairs and the distractor was slightly different (see Figure 4). In the main experiment, no blank was presented between the original image and the modified image, and each image was presented for 1,000 ms. In the binocular and monocular conditions, the AR image was presented for the first 250 ms of the presentation of the original or modified image. The duration of the images seemingly became longer than the duration in the preliminary study (750 ms). However, participants could observe the images without any distractions only for the same duration as the preliminary study because the AR distractor was presented for 250 ms.

The AR image was presented by using the semi-transparent mirror and the LCD. By rotating the polarized filter holders, an experimenter could manipulate whether an AR image was presented to a particular eye. Thus, by using the polarized filter holders, the AR image was presented either binocularly or monocularly. In the monocular condition, the AR image was presented to participants’ right eye. In the none condition, the AR image was not presented; so, participants could observe the moment of the change without any obstruction. In the binocular and monocular conditions, 12 pairs of images were presented in each luminance condition. The order of the trial was randomized. In the none condition, there was no luminance condition because the AR image was not presented. Therefore, all 36 pairs of images were presented without the distractor in the none condition.

A set of 12 image pairs was assigned to each luminance condition. This assignment was counterbalanced between the participants.

Three practice trials were conducted before starting the main experiment. In the practice trial, each change condition was presented once.

#### Experimental Design

The number of trials was determined in accordance with the following formula: 3 luminance conditions (high, medium, low) × 12 (4 times each for change of color, location, disappearing), meaning 36 trials for each participant. The observation condition (3: binocular, monocular, none) was a between design.

The time between the starting point of the stimuli presentation and pressing the 5 key was recorded as the reaction time. Subjective conspicuity and predictivity were also recorded.

#### Results

The binocular and monocular conditions cannot be directly compared with the none condition because there was no luminance condition in the none condition. Hence, first, we analyzed each data using a 2 (observation condition: binocular, monocular) × 3 (luminance condition: high, medium, low) mixed design analysis of variance (ANOVA). All degrees of freedom were adjusted by Chi-Muller’s ɛ, and Shaffer’s procedure was used in all multiple comparisons.

We measured the dominant eye of participants, but Kitamura et al. (2015) revealed that there was no significant difference between the situations in which the AR was presented to the dominant or non-dominant eye. Moreover, we conducted ANOVA including the eye-dominance factor and also found and also found no significant difference between dominant and non-dominant eye participants. Hence, we decided not to include eye dominance as factor in these experiments.

A Welch two-sample test was conducted to compare the none condition with the binocular-high luminance, binocular-medium luminance, binocular-low luminance, monocular-high luminance, monocular-medium luminance, and monocular-low luminance conditions. The level of significance was adjusted by the Bonferroni procedure. In other words, 0.05/6 = 0.00833… was treated as an adjusted level of significance, and when *p* was below this value, we consider the result as significant.

We did not conduct statistical analysis for the error rate or accuracy of clicked location for two reasons. First, participants pressed the key after they fully recognized where or what was changed, and in the phase of clicking, they could observe the stimuli image without any distractors with both eyes not only in the binocular condition but also in monocular and none conditions. Therefore, there must have been no difference among the conditions in terms of accuracy of the clicked place. Second, the error rate of the clicked location was extremely low (1.4% among all trials), which revealed that participants could detect changes correctly in almost all trials at least before the end of the trial. These means that meaningful statistical analysis cannot be conducted.

##### Alternation Times

In the main experiment, the images were alternated every 1,000 ms. Therefore, we discarded the data in which the reaction time was less than 1,000 ms because it was a response made before the first alternation (9/1,296 trials, 0.7%). The data in which the participant could not click the correct location of the change were also excluded from the analysis (18/1,287 trials, 1.4%).

The remaining data were transformed from the reaction time to the number of alternations. For example, if the reaction time was between 1,000 and 1,999 ms, the value was transformed to one alternation, and between 2,000 and 2,999 ms, the value was two alternations. After this transformation, we calculated mean alternation times and a SD for each participant and luminance condition, and we excluded the data that exceeded mean ± 2 SD (82/1,269 trials, 6.5%).

**Figure 5:**
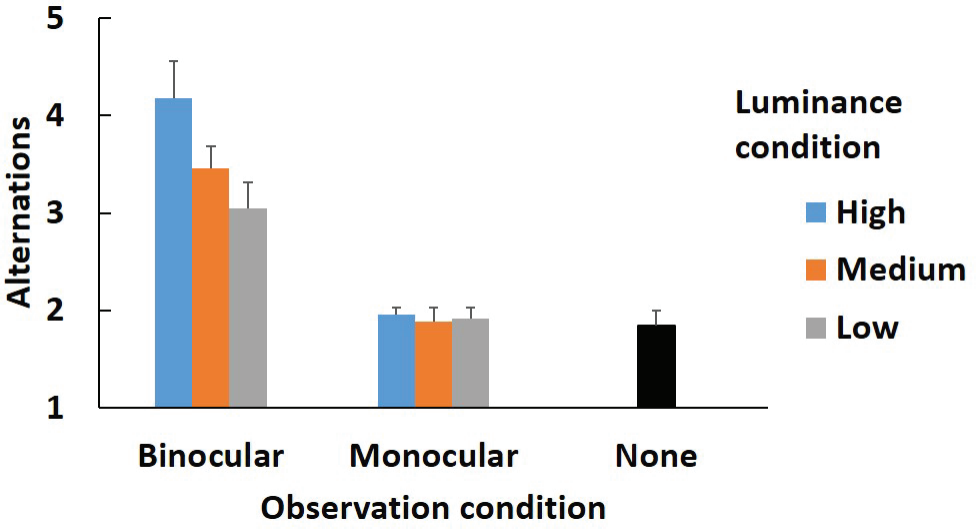
Alternation times in main experiment. Error bars indicate standard error.

Figure 5 shows the alternations in each condition in the main experiment. We analyzed the alternations using a 2 (observation conditions) × 3 (luminance conditions) mixed design ANOVA.

The main effects of the observation and luminance conditions [*F*(1, 22) = 41.68, *p* < 0.001, *η*^2^ = 0.503; *F*(2, 44) = 6.54, *p* < 0.01, *η*^2^ = 0.045] and the interaction between them [*F*(2, 44) = 5.54, *p* < 0.01, *η*^2^ = 0.038] were all significant. The analysis (the observation condition × the luminance condition) showed that the simple main effect of the observation condition was significant in all luminance conditions [high, *F*(1, 22) = 33.55, *p* < 0.001, *η*^2^ = 0.604; medium, *F*(1, 22) = 35.99, *p* < 0.001, *η*^2^ = 0.621; low, *F*(1, 22) = 14.64, *p* < 0.001, *η*^2^ = 0.400]. There were fewer alternations in all luminance conditions in the monocular condition than in the binocular condition. The simple main effect of the luminance was significant only in the binocular condition [binocular, *F*(2, 22) = 6.31, *p* < 0.01, *η*^2^ = 0.184; monocular, *F*(1.62, 17.77) = 0.44, *p* > 0.05, *η*^2^ = 0.005]. Multiple comparisons showed that there were more alternations in the high luminance condition (*M* = 4.18, SD = 1.31) than in the medium (*M* = 3.46, SD = 0.77) and low conditions (*M* = 3.05, SD = 0.94) (*p* < 0.05). The difference between the medium and low conditions was not significant (*p* > 0.05).

A Welch two-sample test was conducted to compare the none condition with each observation and luminance condition. The results are shown in Table 1. After adjustment by the Bonferroni procedure, the differences between the binocular and none conditions in all luminance conditions were significant (adj. *p* < 0.05) but those between all the monocular and none conditions were not (adj. *p* > 0.05).

**Table 1:** Comparison with the none condition in main experiment.

	Alternation times			Conspicuity			Predictivity		
Condition	*t*	*df*	Cohen’s *d*	*t*	*df*	Cohen’s *d*	*t*	*df*	Cohen’s *d*
Bino/high	5.74^*^	14.49	2.34	0.36	21.83	0.15	1.88	22.00	0.77
Bino/medium	6.01^*^	19.51	2.46	0.63	20.93	0.26	2.40	21.62	0.98
Bino/low	3.85^*^	17.26	1.57	1.61	20.39	0.66	2.36	21.98	0.96
Mono/high	0.63	15.98	0.26	2.55	20.62	1.04	2.19	21.86	0.89
Mono/medium	0.20	21.87	0.08	1.89	22.00	0.77	2.09	19.30	0.85
Mono/low	0.35	20.56	0.14	1.45	21.87	0.59	2.23	19.12	0.91

*Bino and mono mean observation conditions. High, medium, and low mean luminance conditions*.

* *p < 0.05 in Bonferroni procedure*.

##### Subjective Conspicuity and Predictivity

**Figure 6:**
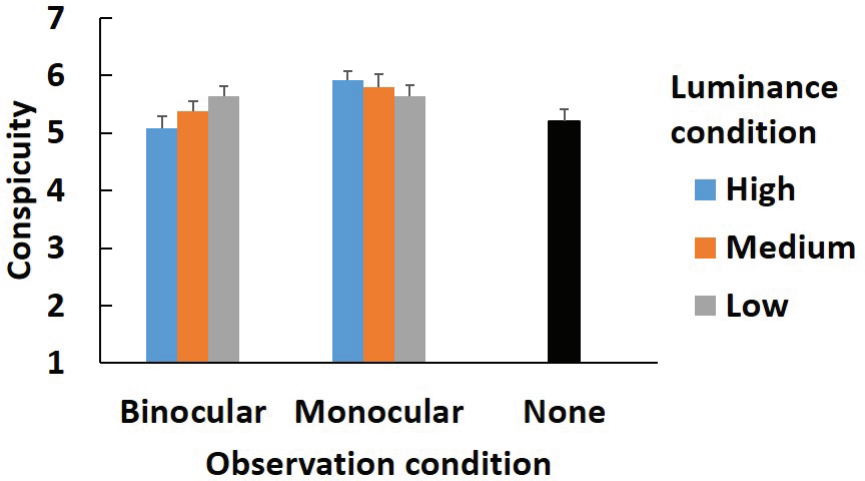
Subjective conspicuity in main experiment. Error bars indicate standard error.

**Figure 7:**
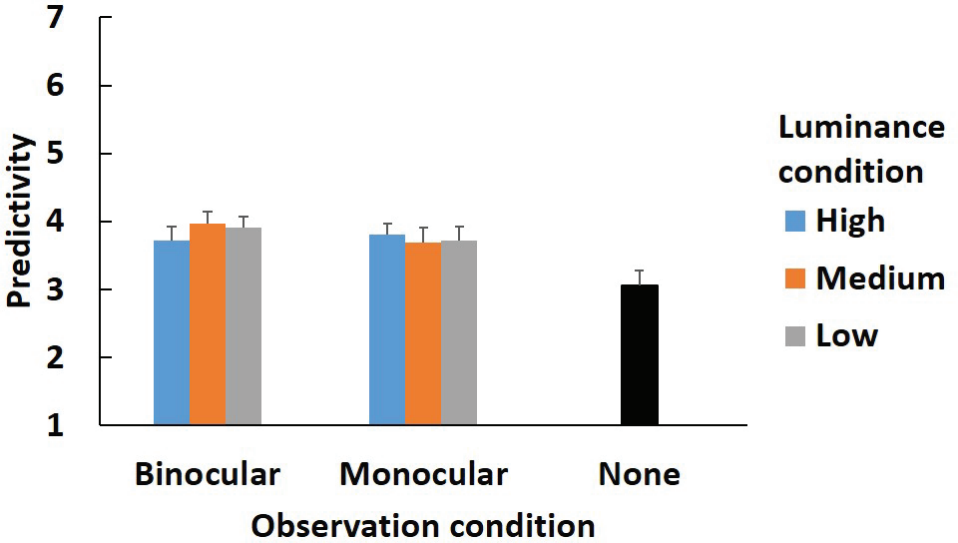
Subjective predictivity in main experiment. Error bars indicate standard error.

Subjective conspicuity and predictivity are also analyzed by using same method as the alternations. Figures 6, 7 show the results.

In terms of conspicuity, the interaction between the observation condition and the luminance condition was significant [*F*(2, 44) = 9.82, *p* < 0.001, *η*^2^ = 0.060]. The main effects of the observation condition and the luminance condition [*F*(1, 22) = 2.71, *p* > 0.05, *η*^2^ = 0.088; *F*(2, 44) = 1.14, *p* > 0.05, *η*^2^ = 0.007] were not significant.

The analysis (the observation condition × the luminance condition) showed that the simple main effect of the observation condition was significant in the high luminance condition [*F*(1, 22) = 9.64, *p* < 0.01, *η*^2^ = 0.305]. The value of the subjective conspicuity was higher in the monocular condition than in the binocular condition. The simple main effect of the luminance condition in the binocular condition was significant [*F*(2, 22) = 10.53, *p* < 0.001, *η*^2^ = 0.123]. Multiple comparisons showed that the conspicuity in the high luminance condition was less than in the medium and low conditions (*p* < 0.05). The difference between the medium and low conditions was not significant (*p* > 0.05).

A Welch two-sample test was conducted to compare the none condition with each observation and luminance condition. The results are shown in Table 1. After adjustment by the Bonferroni procedure, there was no significant difference between the none condition and any observation and luminance conditions (adj. *p* > 0.05).

In the predictivity, the main effects of the observation and luminance conditions and the interaction between them were not significant [observation condition, *F*(1, 22) = 0.19, *p* > 0.05, *η*^2^ = 0.007; luminance condition, *F*(1.99, 43.78) = 0.15, *p* > 0.05, *η*^2^ = 0.001; interaction, *F*(1.99, 43.78) = 1.14, *p* > 0.05, *η*^2^ = 0.010].

A Welch two-sample test was conducted to compare the none condition with each observation and luminance condition. The results are shown in Table 1. After adjustment by the Bonferroni procedure, there was no significant difference between the none condition and any observation and luminance conditions (adj. *p* > 0.05).

## Discussion

In the present study, to investigate change blindness when AR is used, a flicker paradigm task was conducted while a distractor was presented binocularly or monocularly. We manipulated the luminance of the AR image to control the intensity of the distractor during the task.

Comparison with the none condition (Table 1) revealed that change blindness occurred much more frequently in the binocular condition. On the other hand, the number of alternations was not significantly different between the monocular and none conditions. Therefore, participants in the monocular condition could detect the change as early as those in the none condition, and this implies that change blindness was apparently avoided in the monocular condition. Hence, this result supports the first hypothesis that change blindness would occur less in the monocular condition than in the binocular condition.

Regarding the luminance condition, the number of alternations needed to detect the change was less in the monocular condition than in the binocular condition in all luminance conditions (Figure 5). Even in the low luminance condition, in which participants could detect the change the earliest in the binocular condition, more alternations were needed to detect the change than in any luminance condition in the monocular condition. Therefore, the result revealed that changes were much easier to detect in the monocular condition than in the binocular condition.

Moreover, in the binocular condition, change blindness occurred more in higher luminance conditions. This result implies that the real world became more difficult to observe due to the AR distractor in the higher luminance condition in the binocular condition because the AR image was less translucent, resulting in attention being less likely to be attracted to the place where the change occurred. In the monocular condition, by contrast, the number of alternations in all luminance conditions was no higher than in the none condition. This result indicates that participants could select information from the eye to which the AR distractor was not presented. Hence, this result supports the second hypothesis that the change blindness in the binocular condition would occur less frequently in the lower luminance condition than in the higher luminance condition.

From the results of subjective conspicuity, the interaction between the observation condition and the luminance condition was significant (Figure 6). In the binocular condition, the subjective conspicuity was lower in the high luminance condition than in the medium and low conditions. On the other hand, the simple main effect of the luminance condition was not significant in the monocular condition. Moreover, the monocular-high luminance condition had a higher score than the binocular-high luminance condition. These results are congruent with the results for alternations. In the low luminance condition, the real world is comparatively easy to observe through the AR distractor even in the binocular condition; hence, participants might feel the amount of the change more. By contrast, in the monocular condition, the subjective amount of the change did not vary with statistical significance among the luminance conditions. This might be because the information from the eye to which the AR distractor was presented was always suppressed, so the luminance condition did not influence the subjective conspicuity.

According to coherence theory (Rensink, 2002), visual representation from before the change can be compared with the visual input after the change only when the attention is directed there. If participants could observe where the change occurred without any distraction, the change itself attracted attention because changes in color or luminance behave as abrupt onsets of stimulus (Posner, 1980; Yantis and Jonides, 1984; Wright and Ward, 2008). Therefore, the visual representation of the location where the change occurred should be held well, and change blindness should not occur.

In the monocular condition, participants could detect the change as early as in the none condition in all luminance conditions. Therefore, it is supposed that visual attention was attracted when the change occurred as if the AR distractor was not presented, and the visual representation of the location of the change was similarly well organized to that in the none condition, so it was very easy to compare the visual representation with input from after the change. Thus, it is implied that the input from the eye to which the AR distractor was presented was suppressed by the other eye, in which only the stimulus in the real world was presented, and only input from the real world was processed to reach the visual representation.

In the binocular condition, it is thought that the attention was hardly ever captured by the location of the change, especially in the high luminance condition, because the AR distractor itself was able to distract from the change. On the other hand, in the lower luminance condition, attention was relatively easily attracted by the location of change, because the AR distractor was more translucent than in the high luminance condition. However, it is supposed that even in the low luminance condition, attention was not as perfectly captured as in the none condition, because there were more alternations in the binocular-low luminance condition than in the none condition. Therefore, it is implied that even though attention was more likely to be captured in the low luminance condition than in the high luminance condition, still the AR image could behave as a distractor.

Change blindness, which is one of the most critical issues in actual AR use, was effectively attenuated in the monocular condition in the present experiment. This characteristic is suitable for safety in actual AR use, so the monocular AR presentation might be an efficient solution to change blindness. However, the AR image used in the present experiment was very different from the AR images used in actual scenario. AR is technology for presenting information, so it basically requires images with meaning and rich colors. Even if a monocular AR image does not have any meaning, if it has rich colors and shapes, it might suppress the visual representation of the real world. In this case, the AR image will be noticed even in the monocular AR presentation, and as a result, change blindness may occur. Moreover, a user must look at the AR information in actual use, whereas the AR used in the present study was just a distractor, so it had to be ignored by participants. When participants have to direct their attention to an AR image, the results might be different, because it is thought that the AR distractor must attract more attention than in the present study. Furthermore, in actual AR use, the AR image may make sharp movements that themselves attract attention, so the influence of movement should be taken into account. In future research, various kinds of distractors (i.e., rich color, edges, meanings, or movement) should be used to investigate whether the superiority of the monocular presentation is still observed in such situations.

Moreover, Kitamura et al. (2014) compared the binocular AR presentation with the monocular AR presentation when there is the depth difference between an AR image and the real world. In this situation, it was revealed that participants could distribute their attention to a wider area in the monocular condition than in the binocular condition. However, this attentional superiority in the monocular AR presentation was not observed when there was no depth difference between the AR image and the real world. Moreover, in the binocular condition, when participants observe the real world, depth difference between an AR image and the real world makes the AR image look doubled. By contrast, this double image never occurs in the monocular condition. Of course, no double image occurs in either the monocular condition or the binocular condition when there is no depth difference between the AR image and the real world. Thus, depth difference would influence the result of comparison between the binocular and monocular presentations. In this study, only the situation in which there was no depth difference between the AR images was investigated, so the depth factor should be tackled in future research.

In addition, considering binocular rivalry, unstable perception will occur in the monocular condition and may cause fatigue that is not felt in the binocular condition. This point was not considered in the present study, so it should be tackled in a future study. If the monocular condition has some disadvantages compared with the binocular condition, the device that can switch from the binocular presentation to the monocular presentation depending on a particular situation or user’s preference may be desirable in actual AR use.

## Conclusion

In conclusion, the monocular AR presentation demonstrated superiority in terms of attenuating change blindness. This result might be because participants could observe the stimuli when a change occurred by using the eye to which the AR image was not presented; hence, their visual attention was attracted to the location where the change had occurred automatically. However, more various AR distractors should be used in the same design experiments to investigate how stable the superiority is. Therefore, monocular AR presentation must be scrutinized more thoroughly in both engineering and psychology before applying the results in this paper to actual scenarios.

## Data Availability

All datasets generated for this study are included in the manuscript and/or the supplementary files.

## Author Contributions

AK and KS contributed to the conception and design the study. AK contributed to the data acquisition. AK and YK contributed to the statistical analysis. AK wrote the first draft of the manuscript. All authors contributed to manuscript discussion, revision, reading, and final approval of the submitted version.

### Conflict of Interest Statement

The authors declare that the research was conducted in the absence of any commercial or financial relationships that could be construed as a potential conflict of interest.
